# Supplementary material for: Identification of rare sequence variation underlying heritable pulmonary arterial hypertension
Source: Nat Commun. 2018 Apr 12;9:1416. doi: 10.1038/s41467-018-03672-4 (PMC5897357; doi:10.1038/s41467-018-03672-4)
Supplement: Supplementary file 5 — Supplementary Information(PDF 394 kb) [file 41467_2018_3672_MOESM5_ESM.pdf]

Identification of rare sequence variation underlying heritable pulmonary arterial hypertension.

Gräf *et al.*

Supplementary Information

Supplementary Table 1 Patient demographics and clinical characteristics by diagnostic group. Drug and anorexigen group (3 dasatinib exposures, 47 anorexigen exposures, 12 amphetamine exposures); 2 patients had multiple exposures. mPAP - mean pulmonary artery pressure, PCWP - pulmonary capillary wedge pressure, CO - cardiac output, KCO - transfer coefficient for carbon monoxide. Data presented as median [IQR] unless stated.

Group	n	Number with a family history of PAH [%]	Number with a *BMPR2* mutation [%]	WGS inferred ethnicity [% European]	Age at diagnosis (years)	Gender: number female [%]	mPAP (mmHg)	PCWP (mmHg)	CO (L/min)	Functional class 1/2/3/4 [%]	KCO (% predicted)
**IPAH**	908	0 [0.0%]	111 [12.2%]	806 [88.8%]	49.7 [37.0 - 64.6]	624 [68.7%]	53 [44.0 - 61.0]	10 [7 - 12]	4.0 [3.2 - 5.0]	18 [2.3%] / 165 [21.1%] / 506 [64.8%] / 92 [11.8%]	72.0 [51.6 - 87.0]
**HPAH**	58	58 [100.0%]	44 [75.9%]	55 [94.8%]	38.1 [30.4 - 53.5]	34 [58.6%]	54 [48.0 - 64.5]	8 [6 - 11]	3.8 [2.8 - 4.8]	0 [0.0%] / 14 [24.1%] / 37 [63.8%] / 7 [12.1%]	75.5 [69.0 - 85.0]
**Drug and anorexigen exposed**	60	0 [0.0%]	5 [8.3%]	53 [88.3%]	52.4 [40.6 - 60.6]	52 [86.7%]	53 [43.8 - 60.0]	9 [6.5 - 12]	4.2 [3.5 - 5.2]	0 [0.0%] / 10 [17.5%] / 40 [70.2%] / 7 [12.3%]	79.0 [64.0 - 92.6]
**PVOD/PCH**	22	2 [9.1%]	0 [0.0%]	20 [90.9%]	52.2 [39.7 - 68.1]	13 [59.1%]	48 [41.0 - 57.0]	10.5 [8 - 12]	3.5 [2.6 - 4.4]	1 [4.8%] / 2 [9.5%] / 14 [66.7%] / 4 [19%]	38.0 [29.6 - 47.0]

Supplementary Table 2 Copy number deletions identified in *BMPR2* (ENST00000374580) in this study. Previously in Girerd *et al.*[1] reported deletions are marked with asterisks.

Combined (quality based)						MANTA		CANVAS		
Chr	start	end	HGVS (genomic)	HGVS (cDNA)	EXON	start	end	start	end	
2	201106312	204901853	chr2:g.201106312_204901853del	c.-2135886_*1477184del	1-13/13	201106432	204901548	201106312	204901853	
2	201822159	205450144	chr2:g.201822159_205450144del	c.-1420039_*2025475del	1-13/13	201822961	205450144	201822159	205449696	
2	202772559	205218660	chr2:g.202772559_205218660del	c.-469639_*1793991del	1-13/13	202772963	205218660	202772559	205218576	
2	202999184	203486009	chr2:g.202999184_203486009del	c.-243014_*61340del	1-13/13	202999402	203485750	202999184	203486009	
2	203223399	203248850	chr2:g.203223399_203248850del	c.-18799_76+6577del	1/13	203223399	203248060	203223420	203248850	
2	203225712	203256467	chr2:g.203225712_203256467del	c.-16486_76+14194del	1/13	203226401	203255921	203225712	203256467	*
2	203228120	203905293	chr2:g.203228120_203905293del	c.-14078_*480624del	1-13/13	203228906	203905293	203228120	203905004	
2	203234119	203242446	chr2:g.203234119_203242446del	c.-8079_76+173del	1/13	203235908	203242364	203234119	203242446	
2	203325317	203332529	chr2:g.203325317_203332529del	c.77-4215_418+117del	2-3/13	203325958	203332529	203325317	203332065	
2	203327217	203331596	chr2:g.203327217_203331596del	c.77-2315_248-646del	2/13	203327217	203331596	203327390	203331395	*
2	203328407	203332511	chr2:g.203328407_203332511del	c.77-1125_418+99del	2-3/13	203329838	203332511	203328407	203332252	
2	203329730	203335416	chr2:g.203329730_203335416del	c.247+28_418+3004del	3/13	203330505	203335416	203329730	203334949	
2	203339329	203392988	chr2:g.203339329_203392988del	c.418+6917_968-2529del	4-7/13	203339329	203392988	203339531	203391830	
2	203341496	203422058	chr2:g.203341496_203422058del	c.418+9084_2866+804del	4-12/13	203342249	203422058	203341496	203421779	
2	203372762	203382260	chr2:g.203372762_203382260del	c.419-5680_622-1285del	4-5/13	203373003	203382260	203372762	203381787	
2	203379275	203389196	chr2:g.203379275_203389196del	c.530-336_967+4272del	5-7/13	203380130	203388919	203379275	203389196	
2	203383741	203388057	chr2:g.203383741_203388057del	c.818_967+3133del	6-7/13	203384088	203387879	203383741	203388057	
2	203386133	203445336	chr2:g.203386133_203445336del	c.967+1209_*20667del	8-13/13	203387039	203444732	203386133	203445336	
2	203403086	203415133	chr2:g.203403086_203415133del	c.1277-3948_1414-2306del	10/13	203403129	203415088	203403086	203415133	
2	203406092	203412701	chr2:g.203406092_203412701del	c.1277-942_1414-4738del	10/13	203406727	203412701	203406092	203412028	*
2	203406461	203414964	chr2:g.203406461_203414964del	c.1277-573_1414-2475del	10/13	203406613	203414964	203406461	203414695	*
2	203411230	203418627	chr2:g.203411230_203418627del	c.1413+4060_1586+1016del	11/13	203411808	203418627	203411230	203418411	
2	203421702	203434798	chr2:g.203421702_203434798del	c.2866+448_*10129del	13/13	203422398	203434798	203421702	203434395	

Supplementary Table 3 Summary of clinical characteristics in patients with mutations in **(a)** the previously reported PAH genes and **(b)** the novel PAH genes identified in this study. FHx - Family history; mPAP - mean pulmonary artery pressure; PCWP - pulmonary capillary wedge pressure; KCO - transfer coefficient for carbon monoxide.

Gene	Number of patients	FHx of PH [%]	WGS inferred ethnicity: European [%]	Age at diagnosis (years)	Gender: female [%]	mPAP (mmHg)	PCWP (mmHg)	Cardiac output (L/min)	Functional class 1 / 2 / 3 / 4 [%]	KCO (% predicted)
**(a) Previously reported PAH disease genes.**										
**No mutation**	802	9 [1.1%]	713 [88.9%]	52.6 [39.1 - 66.5]	562 [70.1%]	51 [42.0 - 60.0]	10 [7.0 - 12.0]	4.1 [3.3 - 5.2]	16 [2.3%] / 142 [20.5%] / 459 [66.2%] / 76 [11.0%]	69.0 [47.2 - 84.0]
BMPR2*	160	44 [27.5%]	149 [93.1%]	39.3 [32.0 - 51.2]	106 [66.2%]	57 [51.0 - 68.2]	10 [7.0 - 12.0]	3.3 [2.7 - 4.0]	2 [1.3%] / 33 [21.9%] / 90 [59.6%] / 26 [17.2%]	82.0 [73.5 - 92.5]
EIF2AK4	14	2 [14.3%]	7 [50.0%]	31.2 [23.4 - 38.6]	7 [50.0%]	50.5 [41.8 - 59.2]	10.5 [7.2 - 12.0]	4.5 [3.0 - 4.9]	0 [0.0%] / 2 [14.3%] / 9 [64.3%] / 3 [21.4%]	33.0 [29.1 - 33.0]
TBX4	14	1 [7.1%]	12 [85.7%]	56.4 [39.9 - 71.8]	9 [64.3%]	58 [48.0 - 67.0]	11 [7.5 - 13.5]	3.8 [2.9 - 4.1]	0 [0.0%] / 2 [18.2%] / 7 [63.6%] / 2 [18.2%]	89.7 [83.3 - 91.8]
ACVRL1	9	1 [11.1%]	9 [100.0%]	46.9 [44.8 - 57.7]	8 [88.9%]	54 [42.8 - 58.5]	11.5 [10.2 - 12.8]	4.6 [3.5 - 5.0]	0 [0.0%] / 4 [57.1%] / 3 [42.9%] / 0 [0.0%]	72.1 [60.5 - 77.3]
ENG	5	0 [0.0%]	4 [80.0%]	42.2 [35.7 - 47.2]	4 [80.0%]	55 [52.0 - 58.0]	6.5 [0.8 - 13.5]	3.8 [3.5 - 4.2]	0 [0.0%] / 0 [0.0%] / 3 [75%] / 1 [25%]	47.0 [46.0 - 51.0]
KCNK3	4	0 [0.0%]	4 [100.0%]	26.8 [25.1 - 38.5]	2 [50.0%]	48 [43.5 - 63.0]	7.5 [6.2 - 8.8]	3.8 [2.3 - 5.1]	0 [0.0%] / 1 [33.3%] / 2 [66.7%] / 0 [0.0%]	85.7 [85.7 - 85.7]
SMAD9	3	0 [0.0%]	2 [66.7%]	51.3 [39.8 - 56.2]	2 [66.7%]	46 [43.0 - 55.0]	11 [9.0 - 12.0]	5.0 [4.7 - 5.0]	0 [0.0%] / 0 [0.0%] / 3 [100%] / 0 [0.0%]	92.5 [81.2 - 103.8]
**(b) Novel PAH disease genes.**										
ATP13A3	11	0 [0.0%]	9 [81.8%]	47.5 [41.5 - 58.4]	10 [90.9%]	57 [49.5 - 62.0]	10.5 [7.5 - 16.0]	3.1 [2.6 - 4.6]	0 [0.0%] / 0 [0.0%] / 8 [88.9%] / 1 [11.1%]	88.5 [82.2 - 91.4]
SOX17	9	0 [0.0%]	9 [100.0%]	27.3 [21.4 - 40.2]	5 [55.6%]	58 [57.0 - 68.0]	10 [7.0 - 11.5]	4.1 [3.8 - 5.1]	1 [14.3%] / 1 [14.3%] / 5 [71.4%] / 0 [0.0%]	80.9 [78.3 - 83.5]
AQP1	9	2 [22.2%]	9 [100.0%]	32.2 [25.3 - 46.2]	4 [44.4%]	61.5 [48.0 - 68.2]	8 [7.5 - 8.0]	4.7 [4.3 - 5.3]	0 [0.0%] / 4 [50%] / 3 [37.5%] / 1 [12.5%]	81 [78.2 - 91.0]
GDF2	8	1 [12.5%]	7 [87.5%]	49.9 [45.0 - 53.8]	4 [50.0%]	46 [43.2 - 50.5]	8 [6.0 - 9.5]	4.8 [3.7 - 4.9]	0 [0.0%] / 2 [28.6%] / 5 [71.4%] / 0 [0.0%]	70.0 [69.0 - 71.1]

* This group includes one case with deleterious variant in *BMPR2* and *SMAD9*.

Supplementary Table 4 Case-control analysis of variants in previously reported PAH genes. Only protein-coding variants were considered after standard filtering and variants shared between cases and controls and variants of uncertain significance in cases have been removed. *P* values have been adjusted for false discovery rate.

Gene	Number of cases	Number of controls	Frequency of cases	Frequency of controls	*P*	Adj. *P*
BMPR2	148	20	0.1426	0.0031	7.77E-107	1.01E-105
EIF2AK4	14	0	0.0135	0	1.01E-12	6.59E-12
TBX4	14	15	0.0135	0.0023	9.99E-06	4.33E-05
ACVRL1	9	12	0.0087	0.0019	0.0012	0.0039
ENG	6	16	0.0058	0.0025	0.0759	0.1973
KCNK3	4	13	0.0039	0.0020	0.2057	0.4386
KLF2	3	10	0.0029	0.0016	0.2697	0.4386
SMAD9*	4	15	0.0039	0.0023	0.2699	0.4386
BMPR1B	4	17	0.0039	0.0027	0.3369	0.4867
TOPBP1	5	25	0.0048	0.0039	0.4119	0.5354
CAV1	0	5	0	8.00E-04	1	1
SMAD1	0	10	0	0.0016	1	1
SMAD4	0	8	0	0.0013	1	1

* This group includes one case with deleterious variant in *BMPR2* and *SMAD9*.

Supplementary Table 5 List of top 50 genes from rare protein truncating variant (PTV) case-control analysis, excluding patients carrying variants in previously reported genes. Genes are ranked by *P* value.

Gene	No. of individuals in cases	No. of individuals in controls	Frequency of cases	Frequency of controls	*P*	adj. *P*
ATP13A3	6	0	0.00715	0.00000	2.42E-06	0.03505
EVI5	5	1	0.00596	0.00016	0.00011	1
KDR	4	0	0.00477	0.00000	0.00018	1
CAMTA2	7	8	0.00834	0.00125	0.00077	1
RAB36	4	1	0.00477	0.00016	0.00082	1
ERI1	4	1	0.00477	0.00016	0.00082	1
KIF1C	4	1	0.00477	0.00016	0.00082	1
ALG10	4	1	0.00477	0.00016	0.00082	1
PRSS53	4	1	0.00477	0.00016	0.00082	1
ZFP69B	4	1	0.00477	0.00016	0.00082	1
LCAT	4	1	0.00477	0.00016	0.00082	1
COMT	3	0	0.00358	0.00000	0.00156	1
SRM	3	0	0.00358	0.00000	0.00156	1
ZMYM5	3	0	0.00358	0.00000	0.00156	1
FRMD1	3	0	0.00358	0.00000	0.00156	1
CYP3A4	3	0	0.00358	0.00000	0.00156	1
METTL7B	3	0	0.00358	0.00000	0.00156	1
JAG2	3	0	0.00358	0.00000	0.00156	1
FAM151A	5	4	0.00596	0.00063	0.00176	1
TARBP1	6	7	0.00715	0.00110	0.00201	1
HAP1	6	7	0.00715	0.00110	0.00201	1
CDK11A	4	2	0.00477	0.00031	0.00223	1
LILRB5	4	2	0.00477	0.00031	0.00223	1
MYH3	4	2	0.00477	0.00031	0.00223	1
IFIH1	7	12	0.00834	0.00188	0.00397	1
SHC2	4	3	0.00477	0.00047	0.00473	1
ALPPL2	4	3	0.00477	0.00047	0.00473	1
CTSF	4	3	0.00477	0.00047	0.00473	1
SUGCT	4	3	0.00477	0.00047	0.00473	1
ATP6V0A2	4	3	0.00477	0.00047	0.00473	1
EXD3	4	3	0.00477	0.00047	0.00473	1
TFAP2E	3	1	0.00358	0.00016	0.00570	1
PIGT	3	1	0.00358	0.00016	0.00570	1
TMEM62	3	1	0.00358	0.00016	0.00570	1
RHOT2	3	1	0.00358	0.00016	0.00570	1
ENAH	3	1	0.00358	0.00016	0.00570	1
KMT2D	3	1	0.00358	0.00016	0.00570	1
ANTXR1	3	1	0.00358	0.00016	0.00570	1
ZFPM1	3	1	0.00358	0.00016	0.00570	1
RXRB	3	1	0.00358	0.00016	0.00570	1
N4BP2L2	3	1	0.00358	0.00016	0.00570	1
SACS	5	8	0.00596	0.00125	0.01208	1
TTC37	5	8	0.00596	0.00125	0.01208	1

COL9A2	3	2	0.00358	0.00031	0.01303	1
UHRF1BP1	3	2	0.00358	0.00031	0.01303	1
PLEKHH3	3	2	0.00358	0.00031	0.01303	1
FMO2	3	2	0.00358	0.00031	0.01303	1
KRT32	3	2	0.00358	0.00031	0.01303	1
CRBN	3	2	0.00358	0.00031	0.01303	1
PCID2	3	2	0.00358	0.00031	0.01303	1

Supplementary Table 6 List of top 50 genes from rare missense variant case-control analysis, excluding patients carrying variants in previously reported genes. Genes are ranked by *P* value.

Gene	No. of individuals in cases	No. of individuals in controls	Frequency of cases	Frequency of controls	*P*	adj. *P*
GDF2	11	5	0.01311	0.00078	1.23E-07	0.00233
AQP1	8	4	0.00954	0.00063	1.03E-05	0.19641
OR8U1	14	28	0.01669	0.00439	0.00017	1
CACNB2	12	21	0.01430	0.00329	0.00019	1
ATP13A5	13	25	0.01549	0.00392	0.00021	1
C5orf42	20	55	0.02384	0.00861	0.00025	1
FMO4	9	12	0.01073	0.00188	0.00030	1
C5AR1	6	4	0.00715	0.00063	0.00033	1
FLNA	15	35	0.01788	0.00548	0.00037	1
PIWIL1	8	10	0.00954	0.00157	0.00048	1
MICU3	8	10	0.00954	0.00157	0.00048	1
ALPPL2	8	10	0.00954	0.00157	0.00048	1
LGR4	8	10	0.00954	0.00157	0.00048	1
DOCK8	19	54	0.02265	0.00846	0.00049	1
UNC13D	15	37	0.01788	0.00579	0.00059	1
C3orf20	9	14	0.01073	0.00219	0.00066	1
THNSL2	7	8	0.00834	0.00125	0.00077	1
CDCA7	7	8	0.00834	0.00125	0.00077	1
BTNL8	4	1	0.00477	0.00016	0.00082	1
CRYZ	4	1	0.00477	0.00016	0.00082	1
COL5A2	16	43	0.01907	0.00673	0.00083	1
MYO5A	12	26	0.01430	0.00407	0.00085	1
MYZAP	5	3	0.00596	0.00047	0.00087	1
TBC1D2	9	15	0.01073	0.00235	0.00095	1
ZNF48	10	19	0.01192	0.00298	0.00107	1
CYP4Z1	6	6	0.00715	0.00094	0.00120	1
ZXDB	6	6	0.00715	0.00094	0.00120	1
KRTAP4-11	6	6	0.00715	0.00094	0.00120	1
ZC3H6	9	16	0.01073	0.00251	0.00134	1
MFRP	10	20	0.01192	0.00313	0.00144	1
CACNA1C	11	24	0.01311	0.00376	0.00145	1
SPTBN1	14	37	0.01669	0.00579	0.00152	1
HIST1H3I	3	0	0.00358	0.00000	0.00156	1
NBPF12	3	0	0.00358	0.00000	0.00156	1
CLEC2A	3	0	0.00358	0.00000	0.00156	1
PADI4	8	13	0.00954	0.00204	0.00163	1
DEAF1	8	13	0.00954	0.00204	0.00163	1
NUP43	5	4	0.00596	0.00063	0.00176	1
MAP7D3	5	4	0.00596	0.00063	0.00176	1
CD3E	5	4	0.00596	0.00063	0.00176	1
PTPRC	9	17	0.01073	0.00266	0.00184	1
CD248	9	17	0.01073	0.00266	0.00184	1
ABCF1	9	17	0.01073	0.00266	0.00184	1

ZNF292	14	38	0.01669	0.00595	0.00186	1
KLC3	11	25	0.01311	0.00392	0.00188	1
ZNF343	7	10	0.00834	0.00157	0.00189	1
DLL4	7	10	0.00834	0.00157	0.00189	1
ADC	7	10	0.00834	0.00157	0.00189	1
DENND2D	7	10	0.00834	0.00157	0.00189	1
HOMEZ	7	10	0.00834	0.00157	0.00189	1

Supplementary Table 7 List of top 50 genes from combined rare PTV and missense variant case-control analysis, excluding patients carrying variants in previously reported genes. Genes are ranked by *P* value.

Gene	No. of individuals in cases	No. of individuals in controls	Frequency of cases	Frequency of controls	*P*	adj. *P*
GDF2	12	6	0.01430	0.00094	5.33E-08	0.00103
AQP1	9	5	0.01073	0.00078	4.30E-06	0.08294
ALPPL2	12	13	0.01430	0.00204	6.86E-06	0.13246
ATP13A3	11	14	0.01311	0.00219	4.64E-05	0.89511
OR8U1	15	30	0.01788	0.00470	9.92E-05	1
IFT74	11	17	0.01311	0.00266	0.00016	1
FLNA	16	36	0.01907	0.00564	0.00017	1
SOX17	8	9	0.00954	0.00141	0.00030	1
ATP13A5	13	27	0.01549	0.00423	0.00038	1
C3orf20	10	16	0.01192	0.00251	0.00039	1
PIWIL1	8	10	0.00954	0.00157	0.00048	1
DOCK8	20	60	0.02384	0.00940	0.00062	1
C5orf42	21	65	0.02503	0.01018	0.00064	1
RABL3	6	5	0.00715	0.00078	0.00066	1
C5AR1	6	5	0.00715	0.00078	0.00066	1
SPTBN1	15	38	0.01788	0.00595	0.00074	1
SLC9A3R1	8	11	0.00954	0.00172	0.00075	1
CD248	10	18	0.01192	0.00282	0.00078	1
BTNL8	4	1	0.00477	0.00016	0.00082	1
UNC13D	16	43	0.01907	0.00673	0.00083	1
CRTAM	5	3	0.00596	0.00047	0.00087	1
OR13C2	5	3	0.00596	0.00047	0.00087	1
ZNF292	15	39	0.01788	0.00611	0.00091	1
FMO4	9	15	0.01073	0.00235	0.00095	1
CACNB2	14	35	0.01669	0.00548	0.00099	1
MAMDC4	19	58	0.02265	0.00908	0.00100	1
COL5A2	16	44	0.01907	0.00689	0.00101	1
MICU3	8	12	0.00954	0.00188	0.00112	1
IRAK4	8	12	0.00954	0.00188	0.00112	1
LGR4	8	12	0.00954	0.00188	0.00112	1
KRTAP4-11	6	6	0.00715	0.00094	0.00120	1
ICK	7	9	0.00834	0.00141	0.00123	1
HES4	7	9	0.00834	0.00141	0.00123	1
ERMP1	13	32	0.01549	0.00501	0.00133	1
MYO5A	12	28	0.01430	0.00439	0.00141	1
CACNA1C	11	24	0.01311	0.00376	0.00145	1
CACNA1A	24	86	0.02861	0.01347	0.00153	1
OTUB1	3	0	0.00358	0.00000	0.00156	1
NBPF12	3	0	0.00358	0.00000	0.00156	1
ZNF343	8	13	0.00954	0.00204	0.00163	1
NUP43	5	4	0.00596	0.00063	0.00176	1
ZNF561	5	4	0.00596	0.00063	0.00176	1
P2RY14	5	4	0.00596	0.00063	0.00176	1

GALT	5	4	0.00596	0.00063	0.00176	1
DEPDC1	9	17	0.01073	0.00266	0.00184	1
INPP4A	9	17	0.01073	0.00266	0.00184	1
ITIH4	9	17	0.01073	0.00266	0.00184	1
SLC1A7	9	17	0.01073	0.00266	0.00184	1
ZC3H6	9	17	0.01073	0.00266	0.00184	1
TBC1D9B	14	38	0.01669	0.00595	0.00186	1

Supplementary Table 8 List of top 50 genes and *P* values identified by case-control comparison using SKAT-O, excluding carriers of deleterious variants in previously reported genes. Genes are ranked by *P* value.

Ensembl Gene	Gene	Genomic Region	*P*	adj. *P*
ENSG00000240583	*AQP1*	7:30951525-30963244	2.15E-10	4.28E-06
ENSG00000235718	*MFRP*	11:119212258-119217223	6.52E-10	1.30E-05
ENSG00000164736	*SOX17*	8:55370699-55372555	3.36E-09	6.69E-05
ENSG00000110881	*ASIC1*	12:50452550-50475432	5.81E-09	0.00012
ENSG00000187801	*ZFP69B*	1:40916634-40929261	1.17E-08	0.00023
ENSG00000163286	*ALPPL2*	2:233271605-233274582	1.53E-08	0.00031
ENSG00000172199	*OR8U1*	11:56143100-56144029	2.05E-08	0.00041
ENSG00000159377	*PSMB4*	1:151372064-151374305	2.56E-08	0.00051
ENSG00000128802	*GDF2*	10:48413578-48416693	3.99E-08	0.00079
ENSG00000168404	*MLKL*	16:74706402-74729655	4.28E-08	0.00085
ENSG00000110324	*IL10RA*	11:117857183-117870356	6.14E-08	0.00122
ENSG00000177398	*UMODL1*	21:43491426-43557730	6.56E-08	0.00131
ENSG00000197405	*C5AR1*	19:47813153-47824087	1.11E-07	0.00220
ENSG00000141837	*CACNA1A*	19:13318127-13617038	1.21E-07	0.00240
ENSG00000068366	*ACSL4*	X:108887258-108926715	1.27E-07	0.00252
ENSG00000257019	*OR13C2*	9:107366952-107367908	1.34E-07	0.00267
ENSG00000174807	*CD248*	11:66082225-66084498	1.84E-07	0.00366
ENSG00000113758	*DBN1*	5:176884434-176899211	2.41E-07	0.00479
ENSG00000004766	*CCDC132*	7:92861781-92987748	2.83E-07	0.00563
ENSG00000132746	*ALDH3B2*	11:67430686-67434406	2.97E-07	0.00590
ENSG00000128254	*C22orf24*	22:32330104-32334052	3.16E-07	0.00628
ENSG00000198755	*RPL10A*	6:35436212-35438527	3.33E-07	0.00661
ENSG00000101384	*JAG1*	20:10620146-10654178	3.51E-07	0.00699
ENSG00000138073	*PREB*	2:27354282-27357289	4.45E-07	0.00885
ENSG00000066056	*TIE1*	1:43766743-43788393	5.00E-07	0.00995
ENSG00000157240	*FZD1*	7:90894196-90896139	5.16E-07	0.01025
ENSG00000115705	*TPO*	2:1418181-1546246	5.49E-07	0.01091
ENSG00000184451	*CCR10*	17:40831571-40833841	5.71E-07	0.01136
ENSG00000125207	*PIWIL1*	12:130827137-130856143	6.11E-07	0.01214
ENSG00000183690	*EFHC2*	X:44008041-44202834	6.13E-07	0.01219
ENSG00000144354	*CDCA7*	2:174219692-174232392	7.30E-07	0.01451
ENSG00000053501	*USE1*	19:17326215-17330622	7.44E-07	0.01480
ENSG00000126773	*PCNXL4*	14:60581431-60600939	7.74E-07	0.01538
ENSG00000188177	*ZC3H6*	2:113033572-113090065	7.74E-07	0.01539
ENSG00000175785	*PRIMA1*	14:94187790-94254064	1.02E-06	0.02027
ENSG00000156414	*TDRD9*	14:104394847-104518419	1.13E-06	0.02249
ENSG00000144895	*EIF2A*	3:150264590-150301698	1.20E-06	0.02394
ENSG00000076258	*FMO4*	1:171288965-171310978	1.30E-06	0.02580
ENSG00000092929	*UNC13D*	17:73824046-73840418	1.45E-06	0.02892
ENSG00000166033	*HTRA1*	10:124221169-124273875	1.64E-06	0.03266
ENSG00000143862	*ARL8A*	1:202103595-202113700	1.69E-06	0.03365
ENSG00000005379	*BZRAP1*	17:56381731-56405281	1.80E-06	0.03576
ENSG00000108950	*FAM20A*	17:66533618-66596807	1.91E-06	0.03805
ENSG00000131126	*TEX101*	19:43910657-43922549	2.17E-06	0.04321

ENSG00000140853	*NLRC5*	16:57054625-57116440	2.31E-06	0.04599
ENSG00000268552	*AL109927.1*	1:28177085-28177255	2.43E-06	0.04825
ENSG00000163216	*SPRR2D*	1:153012604-153012822	2.53E-06	0.05039
ENSG00000130382	*MLLT1*	19:6213053-6279795	2.53E-06	0.05040
ENSG00000196782	*MAML3*	4:140640477-141074481	2.65E-06	0.05268
ENSG00000196911	*KPNA5*	6:117002498-117053486	2.70E-06	0.05373

Supplementary Table 9 PCR primers for human.

**Target**	**Forward**	**Reverse**
**Quantitative RT-PCR**		
ACTB	GCACCACACCTTCTACAATGA	GTCATCTTCTCGCGGTTGGC
AQP1	CCAGGTGGAGGAGTATGACCT	GACCCCTTCTATTTGGGCTTCA
ATP13A3	GAAAATAGGCACAGGATCAG	ATTTTACACTATGGTGGGTG
B2M	CTCGCGCTACTCTCTCTTTCT	CATTCTCTGCTGGATGACGTG
HPRT	GCTATAAATTCTTTGCTGACCTGCTG	AATTACTTTTATGTCCCCTGTTGACTGG
SOX17	GGACCGCACGGAATTTGAAC	GGACACCACCGAGGAAATGG
**Familial segregation analysis**		
Family II (Figure 4a)		
BMPR2ex1	GTGATACGGGCAGGATCAGT	ATTAAAGGCGATTTCCCTGG
BMPR2ex2	GTCATTCGGATAAGACAAAG	TTTAACATACTCCCATGTCC
BMPR2ex3	TAGCTTACACGTACTCTCAC	CCTGGCTTCAACCTTGAATG
BMPR2ex4	GGGTACAGCCTTTCTAAAGG	GATACTATTGAGGCTGGGTG
BMPR2ex5	GCTGCTAATCTTTCTGCAGC	GAATGAAGTCACTGTTCCAG
BMPR2ex6	CAGAGAGCTGTAGCATTCTG	AAGTGATCCACCTGCCTTAG
BMPR2ex7	TTTGCAAATTCTTTATAAGGATGC	AAAATTTCCTGTTGTGAATTTTGA
BMPR2ex8	TGTTCAATAGTCCCTTTTATTCATTG	CACCTGGCCAGTAGATGTTTT
BMPR2ex9	TGTTCTTCAGAATATGCTACGTTCTC	CTAATTTGCATCCTGCTGCT
BMPR2ex10	GCCTGAAGGGGATGAAAAA	TTGTGGCATTAGGCAACTCC
BMPR2ex11	CATGTGGTAAACTGAAAAGCTCA	TTCTTTGTTGGGTCTCAGTTTCT
BMPR2ex12a	TCAGAGGTGTTAAATTTGGAGAGA	TGAGTGGGTAAAGCAAGCTAGA
BMPR2ex12b	TGGAAACCAACAAGCTAGACC	TCTACCTGCCACACCATTCA
BMPR2ex12c	CTTGAAACAAGTCGAAACTGGA	TAAATGGCCCCAAAAGACAC
BMPR2ex13	CACCCTCCTGAGACATTGGT	GGGTGCTGACAGGAGGATAA
ACVRL1ex2	CTCTGTGATTTCCTCTGGGCA	TACATTCTCCCCAGCTTCTCAA
ACVRL1ex3	AGCTGGGACCACAGTGGCTGA	AGGGCAGGGGCCAAGAAGAT
ACVRL1ex4	AGCTGACCTAGTGGAAGCTGA	CTGATTCTGCAGTTCCTATCTG
ACVRL1ex5	AGGAGCTTGCAGTGACCCAGCA	ATGAGAGCCCTTGGTCCTCATCCA
ACVRL1ex6	AGGCAGCGCAGCATCAAGAT	AAACTTGAGCCCTGAGTGCAG
ACVRL1ex7	TGACGACTCCAGCCTCCCTTAG	CAAGCTCCGCCCACCTGTGAA
ACVRL1ex8	AGGTTTGGGAGAGGGGCAGGAGT	GGCTCCACAGGCTGATTCCCCTT
ACVRL1ex9	TCCTCTGGGTGGTATTGGGCCTC	CAGAAATCCCAGCCATGAGCCAC
ACVRL1ex10	TCTCCTCTGCACCTCTCTCCCAA	CTGCAGGCAGAAAGGAATCAGGTGCT

ENGex1	TCCCTGTGTCCACTTCTCCT	GAATACTTGGGGCCTGGTC
ENGex2	CAGGAAAGCCGTTAGCTCAT	ATCTGCCTTGGAGCTTCCTC
ENGex3	TGGGTGGCACAACCTATACA	TGACCCACAGAGATGGACAG
ENGex4	AATGGGCTGACTCCACAAAT	GGAGCTCAGATTCCTCCTGA
ENGex5	GGGGCTCTGTTAGGTGCAG	GCTTTATAAGGGACCGGAGA
ENGex6	ATCCCATAAACCCACACCTG	CTTCCCTGATCCAGAGGTTG
ENGex7	GTGGGGACACAGCAGGAC	GGTGCTTCACCAACAGTGTG
ENGex8	GGGCACACAGTGATCACACA	GGGCTAGGACCCCAAGAGT
ENGex9	GGTTGTGGTCAGTCCTTGGT	CTGCTCTCCCAAACACACCT
ENGex10	ATCTGCAAAAATGGGCGTAT	TTCCAGACACACATGGCTTG
ENGex11	GAGTCAGGCAACTCCACAGG	AGAAAGGCGGAGAGGAAGTT
ENGex12	CCGATATTTGAAGGCAGCAG	TTCCTGCAAACCACAGACCT
ENGex13	GCCAGGGAGTAAACCTGGA	CCCTTGCCATGTGCTATGT
ENGex14	CAGAGAAGTCGAGGGTCCAT	CAATCCCTCAGAGGCTTCAC
ENGex15	GTGAAGCCTCTGAGGGATTG	GGTGAGTTCACACCAGTGCT
SOX17ex1	ACAGGCCAGAACACGGG	CCTGGTTTTGGGCGTCAG
SOX17ex2a	GTTGCGCAATTCAAAGTC	TAGTACACGTGAAGGGCGC
SOX17ex2b	GACCCGGCTTTCTTCGC	TGTTCAAGTGGCAGACAAAA
Family E011942 (Figure 4b)		
AQP1_ex3a	TATGACTCTCTGCCTTCGC	CCCGTGGGTCTCCTAC
Family E012415 (Figure 4c)		
AQP1_ex2a	GAGGCCTGGGTATCCTTGG	CGCCATCCTCCACCCAAT
Family E010634 (Figure 4d)		
AQP1_ex3b	CCCCACCCTGATTGTTCTCT	CCAGAACAGGAAGGACACT

References

1. Girerd B, Montani D, Jais X, Eyries M, Yaici A, Sztrymf B, et al. Genetic counselling in a national referral centre for pulmonary hypertension. Eur Respir J. 2016 Feb;47(2):541-52

2. den Dunnen JT, Dalgleish R, Maglott DR, Hart RK, Greenblatt MS, McGowan-Jordan J, et al. HGVS Recommendations for the Description of Sequence Variants: 2016 Update. Hum Mutat. 2016 Jun;37(6):564-9

NIHR BioResource Collaborators

NIHR BioResource – Rare Disease Consortium[1], Julian Adlard[2], Munaza Ahmed[3], Tim Aitman[4,5], Hana Alachkar[6], David Allsup[7], Jeff Almeida-King[8], Philip Ancliff[9], Richard Antrobus[10], Ruth Armstrong[11,12,13], Gavin Arno[14,15], Sofie Ashford[1,16], William Astle[1,16,17], Anthony Attwood[1,16], Chris Babbs[18,19], Tamam Bakchoul[20], Tadbir Bariana[21,22], Julian Barwell[23,24], David Bennett[25], David Bentley[26], Agnieszka Bierzynska[27], Tina Biss[28], Marta Bleda[29], Harm Bogaard[30], Christian Bourne[26], Sara Boyce[31], John Bradley[1], Gerome Breen[32,33], Paul Brennan[34,35], Carole Brewer[36], Matthew Brown[1,16], Michael Browning[37], Rachel Buchan[38,39], Matthew Buckland[40], Teofila Bueser[41,42,43], Siobhan Burns[40], Oliver Burren[29], Paul Calleja[44], Gerald Carr-White[42], Keren Carss[1,16], Ruth Casey[11,12,13], Mark Caulfield[45], John Chambers[46,47], Jennifer Chambers[48,49], Floria Cheng[49], Patrick F Chinnery[1,50,51], Martin Christian[52], Colin Church[53], Naomi Clements Brod[1,16], Gerry Coghlan[40], Elizabeth Colby[27], Trevor Cole[54], Janine Collins[55], Peter Collins[56], Camilla Colombo[26], Robin Condliffe[57], Stuart Cook[38,58,59,60], Terry Cook[61], Nichola Cooper[62], Paul Corris[63,64], Abigail Crisp-Hihn[1,16], Nicola Curry[65], Cesare Danesino[66], Matthew Daniels[67,68], Louise Daugherty[1,16], John Davis[1,16], Sri V V Deevi[1,16], Timothy Dent[68], Eleanor Dewhurst[1,16], Peter Dixon[48], Kate Downes[1,16], Anna Drazyk[69], Elizabeth Drewe[70], Tina Dutt[71], David Edgar[72], Karen Edwards[1,16], William Egner[73], Wendy Erber[74], Marie Erwood[1,16], Maria C Estiu[75], Gillian Evans[76], Dafydd Gareth Evans[77], Tamara Everington[78], Mélanie Eyries[79], Remi Favier[80,81,82], Debra Fletcher[1,16], James Fox[1,16], Amy Frary[1,16], Courtney French[83], Kathleen Freson[84], Mattia Frontini[1,16], Daniel Gale[85], Henning Gall[86], Claire Geoghegan[26], Terry Gerighty[26], Stefano Ghio[87], Hossein-Ardeschir Ghofrani[62,86], Simon Gibbs[38], Kimberley Gilmour[88], Barbara Girerd[89,90,91], Sarah Goddard[92], Keith Gomez[21,22], Pavels Gordins[93], David Gosal[6], Stefan Gräf[1,16,29], Luigi Grassi[1,16], Daniel Greene[1,16,17], Lynn Greenhalgh[94], Andreas Greinacher[95], Paolo Gresele[96], Philip Griffiths[97,98], Sofia Grigoriadou[99], Russell Grocock[26], Detelina Grozeva[11], Scott Hackett[100], Charaka Hadinnapola[29], William Hague[101], Matthias Haimel[1,16,29], Matthew Hall[70], Helen Hanson[94], Kirsty Harkness[102], Andrew Harper[38,67,103], Claire Harris[64], Daniel Hart[55], Ahamad Hassan[104], Grant Hayman[105], Alex Henderson[106], Jonathan Hoffmann[54], Rita Horvath[107,108], Arjan Houweling[30], Luke Howard[38], Fengyuan Hu[1,16], Gavin Hudson[107], Joseph Hughes[26], Aarnoud Huissoon[100], Marc Humbert[89,90,91], Sean Humphray[26], Sarah Hunter[26], Matthew Hurles[109], Louise Izatt[110], Roger James[1,16], Sally Johnson[111], Stephen Jolles[112,113], Jennifer Jolley[1,16], Neringa Jurkute[14,22], Mary Kasanicki[114], Hanadi Kazkaz[115], Rashid Kazmi[31], Peter Kelleher[39], David Kiely[57], Nathalie Kingston[1], Robert Klima[44], Myrto Kostadima[1,16], Gabor Kovacs[116,117], Ania Koziell[118,119], Roman Kreuzhuber[1,16], Taco Kuijpers[120,121], Ajith Kumar[3], Dinakantha Kumararatne[114], Manju Kurian[122,123], Michael Laffan[124,125], Fiona Lalloo[77], Michele Lambert[126,127], Hana Lango Allen[1,16], Allan Lawrie[128], Mark Layton[124], Claire Lentaigne[124,125], Adam Levine[85], Rachel Linger[1,16], Hilary Longhurst[99], Eleni Louka[18,19], Robert MacKenzie Ross[129], Bella Madan[130], Eamonn Maher[11,131], Jesmeen Maimaris[88], Sarah Mangles[132], Rutendo Mapeta[1,16], Kevin Marchbank[64], Stephen Marks[9], Hugh S Markus[69], Hanns-Ulrich Marschall[133], Andrew Marshall[134,135,136], Jennifer Martin[1,16,29], Mary Mathias[137], Emma Matthews[22,138], Heather Maxwell[139], Paul McAlinden[64], Mark McCarthy[19,103,140], Stuart Meacham[1,16], Adam Mead[141], Karyn Megy[1,16], Sarju Mehta[142], Michel Michaelides[14], Carolyn Millar[124,125], Shahin Moledina[9], David Montani[89,90,91], Tony Moore[14,15], Nicholas Morrell[1,29], Monika Mozere[85], MPGN/C3 Glomerulopathy Rare Renal Disease group[143], Keith Muir[144], Andrew Mumford[145,146], Michael Newnham[29], Jennifer O'Sullivan[130], Samya Obaji[56], Steven Okoli[18,19], Andrea Olschewski[116], Horst Olschewski[116,117], Kai Ren Ong[54], Elizabeth

Ormondroyd[67,68], Willem Ouwehand[1,16], Sofia Papadia[1,16], Soo-Mi Park[12,13,147], David Parry[5], Joan Paterson[11,12,13], Andrew Peacock[53], John Peden[26], Kathelijne Peerlinck[84], Christopher Penkett[1,16], Joanna Pepke-Zaba[148], Romina Petersen[1,16], Angela Pyle[107], Stuart Rankin[44], Anupama Rao[9], F Lucy Raymond[1,11], Paula Rayner-Matthews[1,16], Christine Rees[26], Augusto Rendon[45], Tara Renton[43], Andrew Rice[149,150], Sylvia Richardson[17], Alex Richter[10], Irene Roberts[18,19,151], Catherine Roughley[76], Noemi Roy[18,19,151], Omid Sadeghi-Alavijeh[85], Moin Saleem[27], Nilesh Samani[152], Alba Sanchis-Juan[1,16], Ravishankar Sargur[73], Simon Satchell[27], Sinisa Savic[153], Laura Scelsi[87], Sol Schulman[154], Marie Scully[115], Claire Searle[155], Werner Seeger[86], Carrock Sewell[156], Denis Seyres[1,16], Susie Shapiro[65], Olga Sharmardina[1,16], Rakefet Shtoyerman[157], Keith Sibson[137], Lucy Side[3], Ilenia Simeoni[1,16], Michael Simpson[158], Suthesh Sivapalaratnam[55], Anne-Bine Skytte[159], Katherine Smith[45], Kenneth G C Smith[29,160], Katie Snape[161], Florent Soubrier[79], Simon Staines[1,16], Emily Staples[29], Hannah Stark[1,16], Jonathan Stephens[1,16], Kathleen Stirrups[1,16], Sophie Stock[1,16], Jay Suntharalingam[129], Emilia Swietlik[29], R Campbell Tait[162], Kate Talks[28], Rhea Tan[69], James Thaventhiran[29], Andreas Themistocleous[25], Moira Thomas[163], Kate Thomson[67,68], Adrian Thrasher[9], Chantal Thys[84], Marc Tischkowitz[164], Catherine Titterton[1,16], Cheng-Hock Toh[71], Mark Toshner[29], Matthew Traylor[69], Carmen Treacy[29,148], Richard Trembath[41], Salih Tuna[1,16], Wojciech Turek[44], Ernest Turro[1,16,17], Tom Vale[25], Chris Van Geet[84], Natalie Van Zuydam[25], Marta Vazquez-Lopez[49], Julie von Ziegenweidt[1,16], Anton Vonk Noordegraaf[30], Quintin Waisfisz[30], Suellen Walker[9], James Ware[38,39,58], Hugh Watkins[67,68,103], Christopher Watt[1,16], Andrew Webster[14,15], Wei Wei[50], Steven Welch[100], Julie Wessels[92], Sarah Westbury[145,146], John-Paul Westwood[115], John Wharton[62], Deborah Whitehorn[1,16], James Whitworth[11,12,13], Martin R Wilkins[62], Catherine Williamson[48,165], Edwin Wong[98], Nicholas Wood[166,167], Yvette Wood[1,16], Geoff Woods[11,114], Emma Woodward[77], Stephen Wort[39,41], Austen Worth[9], Katherine Yates[1,16,29], Patrick Yong[168], Tim Young[1,16], Ping Yu[1,16], Patrick Yu-Wai-Man[50]

Affiliations

[1]NIHR BioResource, Cambridge University Hospitals NHS Foundation, Cambridge Biomedical Campus, Cambridge, UK. [2]Chapel Allerton Hospital, Leeds Teaching Hospitals NHS Trust, Leeds, UK. [3]North East Thames Regional Genetics Service, Great Ormond Street Hospital for Children NHS Foundation Trust, London, UK. [4]MRC Clinical Sciences Centre, Faculty of Medicine, Imperial College London, London, UK. [5]Institute of Genetics and Molecular Medicine, University of Edinburgh, Edinburgh, UK. [6]Salford Royal NHS Foundation Trust, Salford, UK. [7]Queens Centre for Haematology and Oncology, Castle Hill Hospital, Hull and East Yorkshire NHS Trust, Cottingham, UK. [8]European Molecular Biology Laboratory, European Bioinformatics Institute (EMBL-EBI), Wellcome Genome Campus, Hinxton, Cambridge, UK. [9]Great Ormond Street Hospital for Children NHS Foundation Trust, London, UK. [10]University Hospitals Birmingham NHS Foundation Trust, Birmingham, UK. [11]Department of Medical Genetics, Cambridge Institute for Medical Research, University of Cambridge, Cambridge Biomedical Campus, Cambridge, UK. [12]Cancer Research UK Cambridge Centre, Cambridge Biomedical Campus, Cambridge, UK. [13]NIHR Cambridge Biomedical Research Centre, Cambridge Biomedical Campus, Cambridge, UK. [14]Moorfields Eye Hospital NHS Foundation Trust, London, UK. [15]UCL Institute of Ophthalmology, University College London, London, UK. [16]Department of Haematology, University of Cambridge, Cambridge Biomedical Campus, Cambridge, UK. [17]MRC Biostatistics Unit, Cambridge Institute of Public Health, University of Cambridge, Cambridge, UK. [18]MRC Molecular Haematology Unit, Weatherall Institute of Molecular Medicine, University of Oxford, Oxford, UK. [19]NIHR Oxford Biomedical Research Centre, Oxford University

Hospitals Trust, Oxford, UK. [20]Center for Clinical Transfusion Medicine, University Hospital of Tübingen, Tübingen, Germany. [21]The Katharine Dormandy Haemophilia Centre and Thrombosis Unit, Royal Free London NHS Foundation Trust, London, UK. [22]University College London, London, UK. [23]Department of Clinical Genetics, Leicester Royal Infirmary, University Hospitals of Leicester, Leicester, UK. [24]University of Leicester, Leicester, UK. [25]The Nuffield Department of Clinical Neurosciences, University of Oxford, John Radcliffe Hospital, Oxford, UK. [26]Illumina Limited, Chesterford Research Park, Little Chesterford, Nr Saffron Walden, UK. [27]Bristol Renal, University of Bristol, Bristol, UK. [28]Haematology Department, Royal Victoria Infirmary, The Newcastle upon Tyne Hospitals NHS Foundation Trust, Newcastle upon Tyne, UK. [29]Department of Medicine, School of Clinical Medicine, University of Cambridge, Cambridge Biomedical Campus, Cambridge, UK. [30]Department of Pulmonary Medicine, VU University Medical Centre, Amsterdam, The Netherlands. [31]Southampton General Hospital, University Hospital Southampton NHS Foundation Trust, Southampton, UK. [32]MRC Social, Genetic & Developmental Psychiatry Centre, Institute of Psychiatry, Psychology & Neuroscience, King's College London, London, UK. [33]NIHR Biomedical Research Centre for Mental Health, Maudsley Hospital, London, UK. [34]Newcastle University, Newcastle upon Tyne, UK. [35]Newcastle upon Tyne Hospitals NHS Foundation Trust, Newcastle upon Tyne, UK. [36]Department of Clinical Genetics, Royal Devon & Exeter Hospital, Royal Devon and Exeter NHS Foundation Trust, Exeter, UK. [37]Department of Immunology, Leicester Royal Infirmary, Leicester, UK. [38]National Heart and Lung Institute, Imperial College London, Royal Brompton Hospital, London, UK. [39]Royal Brompton Hospital, Royal Brompton and Harefield NHS Foundation Trust, London, UK. [40]Royal Free London NHS Foundation Trust, London, UK. [41]King's College London, London, UK. [42]Guy's and St Thomas' Hospital, Guy's and St Thomas' NHS Foundation Trust, London, UK. [43]King's College Hospital NHS Foundation Trust, London, UK. [44]High Performance Computing Service, University of Cambridge, Cambridge, UK. [45]Genomics England Ltd, London, UK. [46]Epidemiology and Biostatistics, Imperial College London, London, UK. [47]Imperial College Healthcare NHS Trust, London, UK. [48]Division of Women's Health, King's College London, London, UK. [49]Women's Health Research Centre, Surgery and Cancer, Faculty of Medicine, Hammersmith Hospital, Imperial College Healthcare NHS Trust, London, UK. [50]Department of Clinical Neurosciences, School of Clinical Medicine, University of Cambridge, Cambridge Biomedical Campus, Cambridge, UK. [51]Medical Research Council Mitochondrial Biology Unit, Cambridge Biomedical Campus, Cambridge, UK. [52]Children's Renal and Urology Unit, Nottingham Children's Hospital, QMC, Nottingham University Hospitals NHS Trust, Nottingham, UK. [53]Golden Jubilee National Hospital, Glasgow, UK. [54]West Midlands Regional Genetics Service, Birmingham Women's and Children's NHS Foundation Trust, Birmingham, UK. [55]The Royal London Hospital, Barts Health NHS Foundation Trust, London, UK. [56]The Arthur Bloom Haemophilia Centre, University Hospital of Wales, Cardiff, UK. [57]Sheffield Pulmonary Vascular Disease Unit, Royal Hallamshire Hospital NHS Foundation Trust, Sheffield, UK. [58]MRC London Institute of Medical Sciences, Imperial College London, London, UK. [59]National Heart Research Institute Singapore, National Heart Centre Singapore, Singapore, Singapore. [60]Division of Cardiovascular & Metabolic Disorders, Duke-National University of Singapore, Singapore, Singapore. [61]Imperial College Renal and Transplant Centre, Hammersmith Hospital, Imperial College Healthcare NHS Trust, London, UK. [62]Department of Medicine, Imperial College London, London, UK. [63]National Pulmonary Hypertension Service (Newcastle), The Newcastle upon Tyne Hospitals NHS Foundation Trust, Newcastle upon Tyne, UK. [64]Institute of Cellular Medicine, Faculty of Medical Sciences, Newcastle University,

Newcastle upon Tyne, UK. [65]Oxford Haemophilia and Thrombosis Centre, The Churchill Hospital, Oxford University Hospitals NHS Trust, Oxford, UK. [66]Department of Molecular Medicine, General Biology, and Medical Genetics Unit, University of Pavia, Pavia, Italy. [67]Department of Cardiovascular Medicine, Radcliffe Department of Medicine, University of Oxford, Oxford, UK. [68]Oxford University Hospitals NHS Foundation Trust, Oxford, UK. [69]Stroke Research Group, Department of Clinical Neurosciences, University of Cambridge, Cambridge Biomedical Campus, Cambridge, UK. [70]Nottingham University Hospitals NHS Trust, Nottingham, UK. [71]The Roald Dahl Haemostasis and Thrombosis Centre, The Royal Liverpool Hospital, Liverpool, UK. [72]Regional Immunology Service, Kelvin Building, Royal Victoria Hospital, Belfast, UK. [73]Sheffield Teaching Hospitals NHS Foundation Trust, Sheffield, UK. [74]Pathology and Laboratory Medicine, University of Western Australia, Crawley, Australia. [75]Ramón Sardá Mother's and Children's Hospital, Buenos Aires, Argentina. [76]Haemophilia Centre, Kent & Canterbury Hospital, East Kent Hospitals University Foundation Trust, Canterbury, UK. [77]Manchester Centre for Genomic Medicine, Saint Mary's Hospital, Manchester, UK. [78]Salisbury District Hospital, Salisbury NHS Foundation Trust, Salisbury, UK. [79]Departement de genetique, Hopital Pitie-Salpetriere, Paris, France. [80]Service d'Hématologie Biologique, Hôpital d'enfants Armand Trousseau, Paris, France, Paris, France. [81]Inserm U1170, Villejuif, France. [82]Assistance Publique-Hôpitaux de Paris, Département d'Hématologie, Hôpital Armand Trousseau, Paris, France. [83]Department of Paediatrics, School of Clinical Medicine, University of Cambridge , Cambridge Biomedical Campus, Cambridge, UK. [84]Department of Cardiovascular Sciences, Center for Molecular and Vascular Biology, University of Leuven, Leuven, Belgium. [85]UCL Centre for Nephrology, University College London, London, UK. [86]University of Giessen and Marburg Lung Center (UGMLC), Giessen, Germany. [87]Division of Cardiology, Fondazione IRCCS Policlinico S. Matteo, Pavia, Italy. [88]UCL Great Ormond Street Institute of Child Health, London, UK. [89]Universite Paris-Sud, Le Kremlin-Bicêtre, France. [90]Service de Pneumologie, DHU Thorax Innovation, Hôpital Bicêtre, Le Kremlin-Bicêtre, France. [91]INSERM U999, LabEx LERMIT, Centre Chirurgical Marie Lannelongue, Le Plessis Robinson, France. [92]University Hospitals of North Midlands NHS Trust, Stoke-on-Trent, UK. [93]East Yorkshire Regional Adult Immunology and Allergy Unit, Hull Royal Infirmary, Hull & East Yorkshire Hospitals NHS Trust, Hull, UK. [94]Department of Clinical Genetics, Liverpool Women's NHS Foundation, Liverpool, UK. [95]Institute for Immunology and Transfusion Medicine, University of Greifswald, Greifswald, Germany. [96]Section of Internal and Cardiovascular Medicine, University of Perugia, Perugia, Italy. [97]Mitochondrial Research Group, Institute of Genetic Medicine, Newcastle University, Newcastle upon Tyne, UK. [98]Institute of Genetic Medicine, Newcastle University, Newcastle upon Tyne, UK. [99]Barts Health NHS Foundation Trust, London, UK. [100]Birmingham Heartlands Hospital, Heart of England NHS Foundation Trust, Birmingham, UK. [101]ARCH: Australian Research Centre for Health of Women and Babies, Robinson Research Institute, Discipline of Obstetrics and Gynaecology, The University of Adelaide, Women's and Children's Hospital, Adelaide, Australia. [102]Department of Neurology, Sheffield Teaching Hospitals NHS Foundation Trust, Sheffield, UK. [103]Wellcome Trust Centre for Human Genetics, University of Oxford, Oxford, UK. [104]Department of Neurology, Leeds Teaching Hospital NHS Trust, Leeds, UK. [105]Epsom & St Helier University Hospitals NHS Trust, London, UK. [106]Northern Genetics Service, The Newcastle upon Tyne Hospitals NHS Foundation Trust, International Centre for Life, Newcastle upon Tyne, UK. [107]Wellcome Centre for Mitochondrial Research, Institute of Genetic Medicine, Newcastle University, Newcastle upon Tyne, UK. [108]John Walton Muscular Dystrophy Research Centre, Institute of Genetic Medicine, Newcastle University, Newcastle upon Tyne, UK. [109]Wellcome Trust

Sanger Institute, Hinxton, Cambridge, UK. [110]Department of Clinical Genetics, Guy's and St Thomas' NHS Foundation Trust, London, UK. [111]Department of Paediatric Nephrology, Great North Children’s Hospital, Newcastle upon Tyne Hospitals NHS Foundation Trust, Newcastle upon Tyne, UK. [112]University Hospital of Wales, Cardiff, UK. [113]Cardiff & Vale University LHB, Cardiff, UK. [114]Addenbrookes Hospital, Cambridge University Hospitals NHS Foundation Trust, Cambridge, UK. [115]University College London Hospitals NHS Foundation Trust, London, UK. [116]Ludwig Boltzmann Institute for Lung Vascular Research, Graz, Austria. [117]Dept of Internal Medicine, Division of Pulmonology, Medical University of Graz, Graz, Austria. [118]Division of Transplantation Immunology and Mucosal Biology, Department of Experimental Immunobiology, Faculty of Life Sciences and Medicine, King’s College London, London, UK. [119]Department of Paediatric Nephrology, Evelina London Children's Hospital, Guy's & St Thomas' NHS Foundation Trust, London, UK. [120]Department of Pediatric Hematology, Immunology, Rheumatology and Infectious Diseases, Emma Children’s Hospital, Academic Medical Center (AMC), University of Amsterdam, Amsterdam, The Netherlands. [121]Department of Clinical Genetics, Academic Medical Center (AMC), University of Amsterdam, Amsterdam, The Netherlands. [122]Molecular Neurosciences, Developmental Neurosciences, UCL Great Ormond Street Institute of Child Health, London, UK. [123]Department of Neurology, Great Ormond Street Hospital for Children NHS Foundation Trust, London, UK. [124]Department of Haematology, Hammersmith Hospital, Imperial College Healthcare NHS Trust, London, UK. [125]Department of Haematology, Imperial College London, London, UK. [126]Division of Hematology, The Children's Hospital of Philadelphia, Philadelphia, USA. [127]Department of Pediatrics, Perelman School of Medicine at the University of Pennsylvania, Philadelphia, USA. [128]Department of Infection, Immunity & Cardiovascular Disease, University of Sheffield, Sheffield, UK. [129]Royal United Hospitals Bath NHS Foundation Trust, Bath, UK. [130]Department of Haematology, Guy's and St Thomas' NHS Foundation Trust, London, UK. [131]Cambridge NIHR Biomedical Research Centre, Cambridge Biomedical Campus, Cambridge, UK. [132]Haemophilia, Haemostasis and Thrombosis Centre, Hampshire Hospitals NHS Foundation Trust, Basingstoke, UK. [133]Wallenberg Laboratory, Department of Molecular and Clinical Medicine, Sahlgrenska Academy, University of Gothenburg, Gothenburg, Sweden. [134]Faculty of Medical and Human Sciences, Centre for Endocrinology and Diabetes, Institute of Human Development, University of Manchester, Manchester, UK. [135]Department of Clinical Neurophysiology, Manchester Royal Infirmary, Central Manchester University Hospitals National Health Service Foundation Trust, Manchester Academic Health Science Centre, Manchester, UK. [136]National Institute for Health Research/Wellcome Trust Clinical Research Facility, Manchester, UK. [137]Department of Haematology, Great Ormond Street Hospital for Children NHS Foundation Trust, London, UK. [138]The National Hospital for Neurology and Neurosurgery, University College London Hospitals NHS Foundation Trust, London, UK. [139]Royal Hospital for Children, NHS Greater Glasgow and Clyde, Glasgow, UK. [140]Oxford Centre for Diabetes, Endocrinology and Metabolism, University of Oxford, Churchill Hospital, Oxford, UK. [141]Centre for Haematology, Department of Medicine, Hammersmith Hospital, Imperial College Healthcare NHS Trust, London, UK. [142]Department of Clinical Genetics, Addenbrookes Hospital, Cambridge University Hospitals NHS Foundation Trust, Cambridge, UK. [143]MPGN/C3 Glomerulopathy Rare Renal Disease group, , UK. [144]Institute of Neuroscience and Psychology, University of Glasgow, Glasgow, UK. [145]School of Cellular and Molecular Medicine, University of Bristol, Bristol, UK. [146]University Hospitals Bristol NHS Foundation Trust, Bristol, UK. [147]East Anglian Regional Genetics Service, Cambridge University Hospitals NHS Foundation Trust, Cambridge, UK. [148]Royal Papworth Hospital

NHS Foundation Trust, Cambridge, UK. [149]Pain Research, Department of Surgery and Cancer, Faculty of Medicine, Imperial College London, London, UK. [150]Chelsea and Westminster Hospital NHS Foundation Trust, London, UK. [151]Department of Paediatrics, Weatherall Institute of Molecular Medicine, University of Oxford, Oxford, UK. [152]Departments of Cardiovascular Sciences and NIHR Leicester Cardiovascular Biomedical Research Unit, University of Leicester, Leicester, UK. [153]The Leeds Teaching Hospitals NHS Trust, Leeds, UK. [154]Beth Israel Deaconess Medical Centre and Harvard Medical School, Boston, USA. [155]Department of Clinical Genetics, Nottingham University Hospitals NHS Trust, Nottingham, UK. [156]Scunthorpe General Hospital, Northern Lincolnshire and Goole NHS Foundation Trust, Scunthorpe, UK. [157]Clinical Genetics Institute, Kaplan Medical Center, Rehovot, Israel. [158]Genetics and Molecular Medicine, King's College London, London, UK. [159]Aarhus University Hospital, Aarhus, Denmark. [160]Cambridge Institute for Medical Research, University of Cambridge, Cambridge Biomedical Campus, Cambridge, UK. [161]Department of Clinical Genetics, St George's University Hospitals NHS Foundation Trust, London, UK. [162]Glasgow Royal Infirmary, NHS Greater Glasgow and Clyde, Glasgow, UK. [163]Gartnavel General Hospital, NHS Greater Glasgow and Clyde, Glasgow, UK. [164]Addenbrooke's Treatment Centre, Addenbrooke's Hospital, Cambridge University Hospitals NHS Foundation Trust, Cambridge, UK. [165]Institute of Reproductive and Developmental Biology, Surgery and Cancer, Hammersmith Hospital, Imperial College Healthcare NHS Trust, London, UK. [166]Department of Molecular Neuroscience, UCL Institute of Neurology, London, UK. [167]UCL Genetics Institute, London, UK. [168]Frimley Park Hospital, NHS Frimley Health Foundation Trust, Camberley, UK.
