# Supplementary material for: Identification of rare sequence variation underlying heritable pulmonary arterial hypertension
Source: Nat Commun. 2018 Apr 12;9:1416. doi: 10.1038/s41467-018-03672-4 (PMC5897357; doi:10.1038/s41467-018-03672-4)
Supplement: Supplementary file 6 — Description of Additional Supplementary Files(PDF 56 kb) [file 41467_2018_3672_MOESM6_ESM.pdf]

Description of Additional Supplementary Files

File Name: Supplementary Dataset 1

Single nucleotide variants (SNVs) and small insertions and deletions (indels) identified in *BMPR2* (ENST00000374580) in this study. Previously in Girerd et al.[1] reported SNVs are marked with asterisks. Abbreviations: HGVS - Human Genome Variation Society nomenclature (http://varnomen.hgvs.org/)[2]; AC - Allele count; AF - Allele frequency; pahaff - Affected PAH subjects; pahidx - unrelated PAH index cases; ExAC - Exome Aggregation Consortium; UK10K - UK10K project; NR - Not reported.

File Name: Supplementary Dataset 2

SNVs and indels identified in other previously reported genes in this study. Transcripts used: ENST00000388922 (*ACVRL1*), ENST00000373203 (*ENG*), ENST00000302909 (*KCNK3*), ENST00000515385 (*SMAD1*), ENST00000379826 (*SMAD9*), ENST00000240335 (*TBX4*). Abbreviations: HGVS - Human Genome Variation Society nomenclature (http://varnomen.hgvs.org/)[2]; AC - Allele count; AF - Allele frequency; pahaff - Affected PAH subjects; pahidx - unrelated PAH index cases; ExAC - Exome Aggregation Consortium; UK10K - UK10K project; NR - Not reported.

File Name: Supplementary Dataset 3

SNVs and indels identified in novel PAH genes in this study. Transcripts used: ENST00000439040 (*ATP13A3*), ENST00000249598 (*GDF2*), ENST00000509504 (*AQP1*), ENST00000297316 (*SOX17*). Abbreviations: HGVS - Human Genome Variation Society nomenclature (http://varnomen.hgvs.org/)[2]; AC - Allele count; AF - Allele frequency; pahaff - Affected PAH subjects; pahidx - unrelated PAH index cases; ExAC - Exome Aggregation Consortium; UK10K - UK10K project

File Name: Supplementary Dataset 4

Non-coding SNVs and indels surrounding previously reported and novel PAH disease genes. Transcripts used: ENST00000374580 (*BMPR2*), ENST00000388922 (*ACVRL1*), ENST00000373203 (*ENG*), ENST00000302909 (*KCNK3*), ENST00000515385 (*SMAD1*), ENST00000379826 (*SMAD9*), ENST00000240335 (*TBX4*), ENST00000439040 (*ATP13A3*), ENST00000249598 (*GDF2*), ENST00000509504 (*AQP1*), ENST00000297316 (*SOX17*). Variants of unknown significance are highlighted in grey. Abbreviations: HGVS - Human Genome Variation Society nomenclature (http://varnomen.hgvs.org/)[2]; AC - Allele count; AF - Allele frequency; pahaff - Affected PAH subjects; pahidx - unrelated PAH index cases; ExAC - Exome Aggregation Consortium.
